# The predictive value of urinary albumin-to-creatinine ratio for coronary artery abnormalities in Kawasaki disease

**DOI:** 10.3389/fped.2025.1583603

**Published:** 2025-06-12

**Authors:** Ye Chen, Wanlin Huang, Miao Hou, Shuhui Wang, Lei Cao, Xuan Li, Haitao Lv

**Affiliations:** ^1^ Department of Cardiology, Children’s Hospital of Soochow University, Suzhou, China; ^2^ Department of Pediatrics, Suzhou Municipal Hospital, Suzhou, China

**Keywords:** Kawasaki disease, UACR, serum Alb, CA abnormalities, acute phase

## Abstract

**Background:**

To explore the application value of the urinary albumin-to-creatinine ratio (UACR) in the predictive of coronary artery (CA) abnormalities in Kawasaki disease (KD) during acute phase.

**Methods:**

This retrospective study included 109 KD patients who were stratified into CA abnormalities and normal CA groups based on echocardiography at one month after KD onset. Clinical, demographic, and laboratory data were analyzed. Urinary microalbumin and urinary creatinine values were collected during the acute phase before high-dose intravenous immunoglobulin (IVIG) therapy, and UACR was calculated.

**Results:**

The 109 patients consisted of 70 males and 39 females. The orrelation analysis revealed no significant associations between UMA and serum albumin (Alb) (*r* = −0.073, *p* = 0.449), or between UACR and serum Alb (*r* = −0.128, *p* = 0.186) in KD patients. Among the 109 patients, 23 (21.1%) developed CA abnormalities. The levels of UACR, CRP, ALT and NT-proBNP were significantly elevated in the CA abnormalities group compared to the normal CA group, while serum Alb and prealbumin (PA) were decreased (*p* < 0.05). Multivariate binary logistic regression analysis identified elevated UACR and reduced serum Alb levels as independent predictors of CA abnormalities (*p* < 0.05). The optimal cutoff values for UACR and serum Alb were 24.1 mg/g and 37.75 g/L, respectively. Combined UACR and serum Alb, the predictive performance improved, with an area under the curve (AUC) of 0.904 (95% CI: 0.848–0.961), a sensitivity of 91.3%, and a specificity of 81.4%.

**Conclusions:**

UACR and serum Alb, assessed during the acute phase of KD, could serve as early biomarkers for CA abnormalities, particularly when analyzed in combination.

## Introduction

Kawasaki disease (KD) is an acute, self-limiting febrile illness of unknown etiology, predominantly affecting children under the age of five (1). Coronary artery (CA) abnormalities are the most common complications of KD, including CA dilation and CA aneurysms (CAAs) (2). Recent years, it has become the leading cause of acquired heart disease in children of developed countries (3). The peak incidence of CA abnormalities occurs during the acute phase of the disease (4). Despite the therapy of high-dose intravenous immunoglobulin (IVIG), approximately 8%–16% patients developed to CA abnormalities during the acute phase (5–8). While most CA abnormalities occurring in the acute phase will disappear over time, some patients' CA abnormalities may persist or progress, leading to stenosis, obstruction, or even acute myocardial infarction (9). It has been confirmed that the primary pathological changes during the acute phase of KD are systemic vasculitis and vascular endothelial injury (10). In addition to causing CA abnormalities, other vascular-rich organs, such as the liver and kidneys, are also susceptible to damage (11). The most common manifestation of urinary system involvement in KD is sterile pyuria, but proteinuria and mild or subclinical renal injury may also occur (12, 13).

Microalbuminuria is traditionally regarded as a marker of early renal injury. But now it is increasingly considered that the increased kidney endothelial permeability associated with microalbuminuria may be a sign of diffuse endothelial dysfunction, which leads to cardiovascular damage (14, 15). It has been reported that cardiovascular diseases with endothelial dysfunction, such as acute coronary syndrome, pulmonary hypertension, and diabetes, are associated with elevated levels of urinary microalbumin (UMA) (16–18), and UMA can serve as an independent risk factor for various cardiovascular events (15, 18). The Urinary Albumin-to-Creatinine Ratio (UACR) corrects for the effects of urine concentration or dilution on random urine microalbumin measurements and is a reliable method widely used in clinical practice to monitor urinary albumin excretion. Numerous studies have shown that the risk of hypertension, cardiovascular disease, and all-cause mortality increases with elevated UACR, even within its normal range (19–21). As a common pediatric vasculitis, there is limited research on urinary microalbumin in KD. Determination of UACR is a simple, inexpensive, and noninvasive method that could be a promising biomarker to identify a high-risk population of CA abnormalities in KD patients.

Serum albumin (Alb) is considered an important marker of inflammation (22, 23), and hypoalbuminemia is common in KD patients. The purpose of this study was to explore the application value of UACR in the predictive of CA abnormalities and its correlation of serum Alb in KD patients, so as to provide a new biomarker for the predictive of CA abnormalities in KD patients in clinic.

## Materials and methods

### Patients

We retrospectively reviewed the medical records of patients with the main diagnosis of KD who were hospitalized at the Children's Hospital of Soochow University from April 2023 to September 2024. The diagnosis of KD in this study was performed according to the diagnostic criteria established by the American Heart Association (AHA) in 2017 (24). This study was approved by the institutional review board of Children's Hospital of Soochow University (No: 2023CS160), and informed consent forms were obtained from the parents of all patients.

A total of 146 patients were diagnosed with KD in our hospital and underwent urinary microalbumin and urinary creatinine tests during this study period. Thirty-seven patients were excluded. Including 2 patients who received initial IVIG treatment in other hospitals before admission, 23 patients who received glucocorticoids treatment prior to admission, 1 patient who showed a second episode of KD, 9 patients who had initiated or completed IVIG treatment when their urine samples were collected during hospitalization, and 2 patients who lacked echocardiogram reports at the 1-month mark. Finally, 109 patients were enrolled in this study.

All patients routinely received echocardiography at admission within 48 h before or after IVIG, at the second week, and at one month (20–40 days) after KD onset. Based on the results of echocardiography at the 1-month mark, the patients were further categorized into CA abnormalities group (*n* = 23) and normal CA group (*n* = 86). All patients received high-dose IVIG and aspirin therapy, as soon as possible after diagnosis.

### Laboratory indicators and detection methods

We collected laboratory indicators of KD patients before IVIG treatment, included complete blood count, biochemical indices, N-terminal pro-B-type natriuretic peptide (NT-proBNP), erythrocyte sedimentation rate (ESR), urinary microalbumin and urinary creatinine. Complete blood count included white blood cells (WBC), neutrophil ratio (*N*%), platelets (PLT), and hemoglobin (Hb). Biochemical indices included alanine aminotransferase (ALT), aspartate aminotransferase (AST), serum Alb, prealbumin (PA), blood urea nitrogen (BUN), serum creatinine (Cr), serum uric acid (UA) and C-reactive protein (CRP).

Urinary microalbumin was measured by immunoturbidimetric method [Ningbo Meikang Biotechnology Co., Ltd, China, Microalbuminuria Kit (Malb)]. Urinary creatinine was detected by enzymatic method (FUJIFILM Wako Pure Chemical Corporation, Japan, L-type Creatinine M). Hitachi fully automated biochemical analyzer 008 was used to measure both urinary microalbumin and urinary creatinine. UACR was calculated using the formula: UACR (mg/g) = urinary microalbumin (mg/L)/urinary creatinine (*μ*mol/L) × 8,840. And the unit “mg/g” represents milligrams of albumin per gram of creatinine (25).

### Diagnostic criteria for CA abnormalities

Pediatric cardiologists in our hospital operated echocardiograms to obtain measurements of coronary artery dimensions, and used Z-scores normalized by body surface area to assess the severity of CA abnormalities. Whether in the right proximal coronary artery, the left coronary aorta, or the left anterior descending branch, CA abnormalities were diagnosed with a Z score calculated using the Dallaire equation (26). One month after the onset of the disease, patients with a maximum Z score of 2.0–2.5 were considered to have CA dilation, and patients with a maximum Z score of ≥2.5 were considered to have CAAs. Patients with CAAs were classified as small aneurysm (2.5 ≤Z-score <5), medium aneurysm (5 ≤*Z*-score <10, and an absolute internal diameter <8 mm), or giant aneurysm (Z-score ≥10, or an absolute internal diameter ≥8 mm) (2). Two pediatric cardiologists who were blinded to the study patients reviewed all the echocardiograms for interobserver reliability.

### Statistical analysis

Data were analyzed using SPSS 26.0 software. The Kolmogorov–Smirnov test was used to assess the normality of the data. Continuous variables with a normal distribution were expressed as mean ± standard deviation $\left(\bar{x}\pm s\right)$, while non-normally distributed continuous variables were expressed as median (interquartile range, IQR) [M (P25–P75)]. For intergroup comparisons, the independent samples *t*-test was used for normally distributed data, and the Mann–Whitney *U* test was applied for non-normally distributed data. Categorical variables were presented as frequencies or percentages (%) and analyzed using the chi-square (*χ*^2^) test. Pearson correlation was used in correlation analysis. Variables identified as statistically significant in univariate analysis were included in a binary logistic regression analysis. Receiver operating characteristic (ROC) curve was constructed and area under the curve (AUC) was calculated. Statistical significance was set at *P* < 0.05.

## Results

### Baseline clinical characteristics

A total of 109 patients (70 males and 39 females) were included in this analysis. The median age was 33 months (IQR: 19–57.5 months). Echocardiographic examinations performed within 48 h before or after IVIG treatment revealed CA abnormalities in 32 patients (29.3%). Among these, 9 cases (28.1%) showed normalization of coronary dimensions at the 1-month follow-up, while 23 cases (71.9%) continued to exhibit CA abnormalities. Specifically, these abnormalities included 16 cases (69.6%) of small coronary artery aneurysms (CAAs), 3 cases (13.0%) of medium CAAs, and 4 cases (17.4%) of coronary dilation; no giant CAAs were detected.

**Figure 1:**
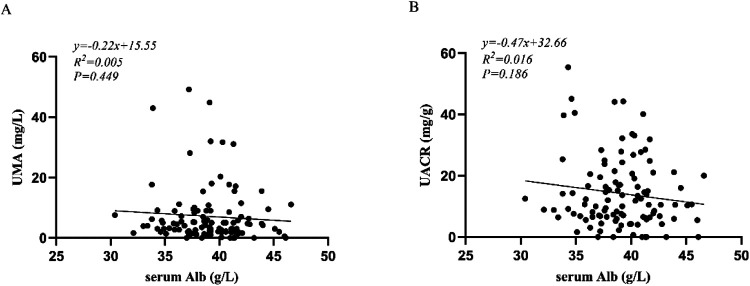
**(A)** correlation analysis between UMA and serum Alb; **(B)** correlation analysis between UACR and serum Alb. UMA, urinary microalbumin; UACR, urinary albumin-to-creatinine ratio; Alb, serum albumin.

The correlation analysis showed that among these 109 patients, there was no significant associations between UMA and serum Alb (*r* = −0.073, *P* = 0.449), between UACR and serum Alb (*r* = −0.128, *p* = 0.186) (Figure 1).

### Univariate analysis of clinical and laboratory indicators in CA abnormalities and normal CA groups

Among the 109 patients, 23 (21.1%) and 86 (78.9%) cases were classified into CA abnormalities and normal CA group, respectively. No statistical differences were observed between the two groups about gender distribution, age or weight (*P* > 0.05). Compared to normal CA group, the CA abnormalities group exhibited significantly higher UACR levels (*P* < 0.05), while UMA levels have no differences between the two groups (*P* > 0.05). Meanwhile, CRP, ALT and NT-proBNP levels were significantly higher (*P* < 0.05), while serum Alb and PA levels were lower (*P* < 0.05) in the CA abnormalities group. However, there were no differences in the levels of WBC, PLT, HB, N%, AST, BUN, serum Cr, or ESR between the groups (*P* > 0.05) (Table 1).

**Table 1:** Comparison of clinical and laboratory characteristics between CA abnormalities group and normal CA group.

Characteristics	CA abnormalities group (*n* = 23)	Normal CA group (*n* = 86)	*P*-value
Males, *n* (%)	18 (78.3)	52 (60.5)	0.114
Age, months	29.00 (20.00–54.00)	33.00 (19.00–58.25)	0.471
Weight, kg	13.00 (11.00–17.00)	14.00 (11.00–18.13)	0.418
UACR, mg/g	25.00 (12.50–32.30)	8.85 (5.58–16.40)	<0.001*
UMA, mg/L	5.70 (3.00–9.20)	3.50 (1.98–7.55)	0.053
WBC, ×10^9^/L	16.41 ± 6.42	14.04 ± 4.88	0.056
Neutrophil ratio, %	66.53 ± 15.84	64.21 ± 16.03	0.539
Hb, ×10^12^/L	110.78 ± 9.31	112.36 ± 8.49	0.440
Platelet, ×10^9^/L	363.39 ± 133.62	342.81 ± 94.43	0.400
Serum Alb, g/L	36.66 ± 2.79	39.71 ± 2.94	<0.001*
AST, U/L	49.60 (30.00–61.60)	33.40 (25.73–50.75)	0.102
ALT, U/L	53.40 (23.90–120.40)	17.50 (11.95–72.55)	0.009*
PA, mg/L	73.00 (65.00–86.00)	84.50 (73.00–98.00)	0.034*
BUN, mmol/L	3.22 ± 1.13	3.32 ± 1.11	0.704
Serum Cr, umol/L	23.40 (20.00–30.20)	29.20 (24.78–32.80)	0.168
UA, umol/L	193.70 ± 54.88	206.37 ± 76.04	0.456
CRP, mg/L	77.82 (49.37–108.63)	54.69 (30.34–80.59)	0.014*
ESR, mm/h	36.83 ± 13.49	42.21 ± 18.52	0.196
NT-proBNP, pg/ml	663.00 (326.40–1,867.00)	268.85 (130.03–677.13)	0.001*

* Indicates a *P* value less than 0.05.

UACR, urinary albumin-to-creatinine ratio; UMA, urinary microalbumin; WBC, white blood cells; Hb, hemoglobin; PLT, platelet; Alb, albumin; AST, aspartate aminotransferase; ALT, alanine aminotransferase; PA, prealbumin; BUN, blood urea nitrogen; Cr, creatinine; UA, serum uric acid; CRP, C-reactive protein; ESR, erythrocyte sedimentation rate; NT-proBNP, N-terminal pro-B-type natriuretic peptide.

### Predictive values of UACR and serum Alb for CA abnormalities in KD

The occurrence of CA abnormalities was designated as the dependent variable, while serum Alb, CRP, ALT, PA, NT-proBNP, and UACR were included as independent variables in the binary logistic regression analysis. It showed that elevated UACR was an independent risk factor for CA abnormalities (*P* < 0.05, OR = 1.123), while serum Alb was a protective factor against CA abnormalities in KD patients (*P* < 0.05, OR = 0.635) (Table 2).

**Table 2:** Multivariate binary logistic regression analysis of the influencing factors for CA abnormalities.

Risk factor	Partial regression coefficient	Standard error	Wald *χ*^2^	*P*-value	OR (95% CI)
Serum Alb	−0.455	0.133	11.629	0.001*	0.635 (0.489–0.824)
UACR	0.116	0.033	12.578	<0.001*	1.123 (1.053–1.197)
CRP	0.004	0.009	0.246	0.620	1.004 (0.998–1.021)
ALT	−0.002	0.002	0.691	0.406	0.998 (0.994–1.003)
PA	0.001	0.012	0.012	0.915	1.001 (0.977–1.026)
NT-proBNP	0.000	0.000	0.001	0.976	1.000 (1.000–1.000)
Constant	13.814	5.214	7.019	0.008*	–

* Indicates a *P* value less than 0.05.

Alb, albumin; UACR, urinary albumin-to-creatinine ratio; CRP, C-reactive protein; ALT, alanine aminotransferase; PA, prealbumin; NT-proBNP, *N*-terminal pro-B-type natriuretic peptide.

**Figure 2:**
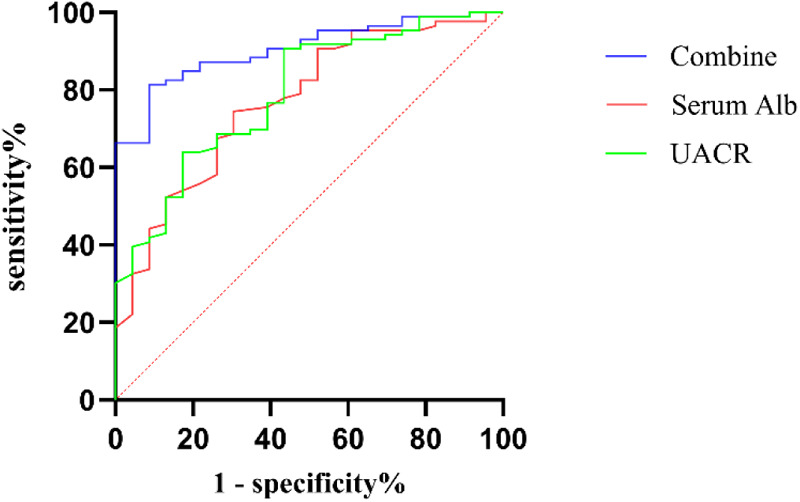
ROC curve of UACR, Serum Alb, and combine diagnosis in CA abnormalities among KD patients. ROC, receiver operating characteristic; Combine, combination of UACR and serum Alb; Alb, serum albumin; UACR, urinary albumin-to-creatinine ratio; CA, coronary artery.

The ROC curves for UACR and serum Alb in the diagnosis of CA abnormalities among KD patients were constructed. For predicting CA abnormalities, the optimal cut-off value of UACR was determined to be 24.1 mg/g, with a sensitivity of 56.5%, specificity of 90.7%, and an AUC of 0.790 (95% CI: 0.691–0.889, *P* < 0.001). The optimal cut-off value of serum Alb was identified as 37.75 g/L, with a sensitivity of 74.4%, specificity of 69.6%, and an AUC of 0.770 (95% CI: 0.665–0.876, *P* < 0.001). When UACR and serum Alb were combined, the predictive performance improved, with an AUC of 0.904 (95% CI: 0.848–0.961, *P* < 0.001), a sensitivity of 91.3%, and a specificity of 81.4% (Figure 2).

## Discussion

The pathological characteristic of KD is immune-mediated vasculitis, which predominantly affects small and medium-sized blood vessels, leading to vascular endothelial damage and dysfunction (27). Without treatment, ≈25% of patients develop coronary artery (CA) dilation or CA aneurysms (CAAs) and in rare cases, thrombosis, stenosis, or occlusion may occur, all of which impact the prognosis of KD patients (2, 7, 8, 28). KD can also lead to injuries in other systems due to systemic vasculitis and vascular endothelial dysfunction. Liver damage is a relatively common occurrence, and alterations in markers of hepatic function such as AST, ALT, serum Alb, and PA have been frequently reported (11, 22, 23). KD can also cause mild or subclinical renal damage. In previous studies, routine urine tests were often used to assess renal damage in KD, but sterile pyuria can originate from both the kidneys and the urethra, making it an imprecise indicator of renal damage (12, 29). Common renal function indicators like BUN and serum Cr are not sensitive to early renal damage. BUN and serum Cr levels are often normal in KD, although BUN may increase in some patients due to dehydration caused by high fever and anorexia (13). Currently, a small number of studies have begun to focus on the significance of urinary microprotein in KD (13, 30, 31). One of the studies found that u-β2MG value were positively associated with CA abnormalities formation, and maximum u-β2MG and maximum coronary diameter before treatment were positively and significantly associated factors (30). UMA is the most common type of urinary microprotein and is not only an important indicator of early renal damage but also a marker of vascular endothelial injury (14, 15). However, there is little reports about UMA in KD, especially its relationship with CA abnormalities.

UMA falls within the category of Alb, which is produced by the liver. Under normal circumstances, it is not easily filtered through the glomerular filtration membrane. However, when the filtration membrane is damaged, and the permeability of the vascular endothelium changes, the charge barrier on its surface is also disrupted. This allows albumin to pass through the glomerular filtration membrane. When the filtration volume exceeds the reabsorption capacity of the proximal tubules, the level of microalbumin in the urine increases, leading to microalbuminuria. The normal range of UMA is 0–20 mg/L, and 20–200 mg/L is defined as microalbuminuria. The 24 h urinary albumin test is the gold standard for evaluating UMA, but collecting 24 h urine is relatively cumbersome and time-consuming, which limits its clinical use. Urinary creatinine can correct for the effects of urine concentration or dilution on UMA levels, making UACR more suitable for reflecting UMA excretion and assessing renal damage in children. Currently, it is recommended to collect a random urine sample, preferably the first morning urine, to detect UACR for diagnosing microalbuminuria, and the UACR of 30 mg/g or higher is defined as renal damage (32). Studies have shown that the risk of cardiovascular diseases increase with an elevated UACR, even within the normal range of less than 30 mg/g, and for every 0.4 mg/mmol increase in URCA, the risk of cardiovascular events increases by 5.9% (19, 33). Urinary albumin excretion is a risk marker for cardiovascular morbidity and mortality, and UACR has been viewed as a marker for vascular endothelial dysfunction in both the kidneys and systemic vasculature (14, 19, 21). UACR is also an inflammatory marker, with its levels correlating with the degree of systemic inflammation (34). Numbers of researches have shown that high-normal UACR can result in the deleterious outcomes. It was reported that by contrast with other biomarkers of endothelial dysfunction and inflammation, only natriuretic peptides and UACR were associated with heart failure patients with preserved ejection fraction (35). The UACR was also higher in dyslipidemia children who has endothelial dysfunction (36). Other researchers have found that worse coronary flow reserve was associated with higher UACR (37), and higher urinary albumin excretion, even within the normal range, is associated with early atherosclerosis in type 1 diabetes during mid-adolescence (38). So UACR can be served as an evaluation indicator in the early stages of many cardiovascular diseases.

Serum Alb is not only a marker reflecting the nutritional status of the body but also a negative acute phase response protein, with its levels inversely correlated with inflammatory responses (2). It is an important inflammatory indicator. KD patients often exhibit varying degrees of hypoalbuminemia, which may be related to various inflammatory cytokines present, such as vascular endothelial growth factor, tumor necrosis factor-α, interleukin-1, and interleukin-6. These cytokines inhibit protein synthesis in the liver and increase vascular permeability, leading to serum Alb leakage and consequently reduced serum Alb levels in the blood (39–41). KD patients with low serum Alb levels tend to experience more severe inflammation, a higher incidence of IVIG resistance, and a higher likelihood of CA abnormalities progression (42–44). Studies have shown that reduced serum Alb levels are an independent risk factor for CA abnormalities in KD (45), and low serum Alb levels have also been proven to be a significant risk factor for CA abnormalities progression (23).

Although albuminuria is considered a major contributor to hypoalbuminemia in many renal diseases, our study found no statistically significant correlation between serum albumin levels and UMA (*r* = −0.073, *p* = 0.449) or UACR (*r* = −0.128, *p* = 0.186) during acute phase of KD. These results suggest that increased urinary microalbumin excretion does not contribute to the pathogenesis of hypoalbuminemia in KD patients. Notably, patients with CA abnormalities exhibited significantly higher UACR levels and lower serum Alb levels compared to those with normal CA. Furthermore, significant differences of CRP, ALT, PA, and NT-proBNP were observed between the two groups, while WBC, PLT, HB, N%, AST, BUN, serum Cr, and ESR were no differences. Multivariable binary logistic regression analysis identified elevated urinary UACR and reduced serum Alb as independent risk factors for CA abnormalities in KD. The optimal cutoff value for UACR was 24.1 mg/g, with a sensitivity of 56.5%, specificity of 90.7%. The optimal cutoff value for serum Alb was 37.75 g/L, with a sensitivity of 74.4%, specificity of 69.6%. When UACR and serum Alb were combined, the diagnostic performance improved, achieving a sensitivity of 91.3%, specificity of 81.4%, and an AUC of 0.904. These findings indicate that the combined use of UACR and serum Alb provides superior predictive accuracy for identifying CA abnormalities in patients with KD.

Our study revealed significantly elevated levels of CRP, ALT, and NT-proBNP, coupled with reduced PA levels, in patients with CA abnormalities compared to those with normal CA. These findings align with previously reported observations (11, 46, 47). However, multivariable logistic regression analysis failed to identify these biomarkers as independent risk factors for CA abnormalities. Our analysis of baseline characteristics—including age, gender, weight, complete blood counts (WBC, PLT, HB, N%)—revealed no statistically significant differences between the two groups. These findings suggested that these variables exert limited influence on the outcomes evaluated in our study.

Limitations of this study include its retrospective design and relatively small sample size. First, as a single-center retrospective study, there may be inherent biases and limitations in data collection and analysis. The relatively modest sample size, though sufficient for initial exploration, may limit the generalizability of our results to broader populations of KD patients. Larger prospective studies are warranted to validate the findings and further elucidate the predictive utility of UACR and its correlation with CA abnormalities in KD. Second, the diversity and variability of treatment status present challenges in controlling for this variable in the current analysi. In future research, we plan to design prospective studies to gather more comprehensive data, including the severity of CA abnormalities, treatment response, and analysis the association between UACR and these clinical parameters. Finally, our study employed the 2017 AHA diagnostic criteria for KD, which had already deemphasized the requirement of “≥5 days of fever” (24). This criterion has also been removed in both the updated 2024 AHA Scientific Statement (2) and the 6th edition of the Japanese Kawasaki Disease Guidelines (48). Notably, while these updated guidelines maintain consistency in diagnostic, they introduce subtle yet clinically significant distinctions in defining coronary artery dilation vs. aneurysms. This evolving diagnostic landscape may affect the comparability of our findings with both previous and future studies.

In summary, we aim to evaluate the clinical utility of UACR as an early biomarker for identifying patients at high risk of developing CA abnormalities. If validated, these findings could enable the early identification of KD patients at heightened risk for CAA during the acute phase, facilitating timely intensification of primary anti-inflammatory therapy. Such an approach may ultimately improve long-term CA outcomes and inform personalized treatment strategies.

## Conclusion

Elevated UACR and hypoalbuminemia independently CA abnormalities in KD patients. The combined use of UACR and serum Alb significantly enhances predictive accuracy for CA abnormalities. Determination of UACR is a simple, inexpensive, and noninvasive method that could be a promising biomarker to identify a high-risk population of CA abnormalities in KD patients. Future studies should expand the sample size to validate the predictive value of these indicators.

These results suggest that urinary microalbumin excretion is unlikely to be the primary mechanism underlying hypoalbuminemia in KD patients.

## Data Availability

The raw data supporting the conclusions of this article will be made available by the authors, without undue reservation.

## References

[1] Kim JM Kim J. Prediction model for the differential diagnosis of kawasaki disease and acute cervical lymphadenitis in patients initially presenting with fever and cervical lymphadenitis. J Pediatr. (2020) 225:30–6.e2. 10.1016/j.jpeds.2020.05.031 32450069

[2] Jone PN Tremoulet A Choueiter N Dominguez SR Harahsheh AS Mitani Y Update on diagnosis and management of Kawasaki disease: a scientific statement from the American Heart Association. Circulation. (2024) 150(23):e481–500. 10.1161/CIR.000000000000129 39534969

[3] Elakabawi K Lin J Jiao F Guo N Yuan Z. Kawasaki disease: global burden and genetic background. Cardiol Res. (2020) 11(1):9–14. 10.14740/cr993 32095191 PMC7011927

[4] Baer AZ Rubin LG Shapiro CA Sood SK Rajan S Shapir Y Prevalence of coronary artery lesions on the initial echocardiogram in Kawasaki syndrome. Arch Pediatr Adolesc Med. (2006) 160(7):686–90. 10.1001/archpedi.160.7.686 16818833

[5] Xie LP Yan WL Huang M Huang MR Chen S Huang GY Epidemiologic features of Kawasaki disease in Shanghai from 2013 through 2017. J Epidemiol. (2020) 30(10):429–35. 10.2188/jea.JE20190065 31548437 PMC7492704

[6] Ae R Makino N Kosami K Kuwabara M Matsubara Y Nakamura Y. Epidemiology, treatments, and cardiac complications in patients with kawasaki disease: the nationwide survey in Japan, 2017–2018. J Pediatr. (2020) 225:23–29.e2. 10.1016/j.jpeds.2020.05.034 32454114

[7] Fukazawa R Kobayashi J Ayusawa M Hamada H Miura M Mitani Y JCS/JSCS 2020 guideline on diagnosis and management of cardiovascular sequelae in Kawasaki disease. Circ J. (2020) 84(8):1348–407. 10.1253/circj.CJ-19-1094 32641591

[8] Wu MH Chen HC Yeh SJ Lin MT Huang SC Huang SK. Prevalence and the long-term coronary risks of patients with Kawasaki disease in a general population <40 years: a national database study. Circ Cardiovasc Qual Outcomes. (2012) 5(4):566–70. 10.1161/CIRCOUTCOMES.112.965194 22589296

[9] Orenstein JM Shulman ST Fox LM Baker SC Takahashi M Bhatti TR Three linked vasculopathic processes characterize Kawasaki disease: a light and transmission electron microscopic study. PLoS One. (2012) 7(6):e38998. 10.1371/journal.pone.0038998 22723916 PMC3377625

[10] Soni PR Noval Rivas M Arditi M. A comprehensive update on Kawasaki disease vasculitis and myocarditis. Curr Rheumatol Rep. (2020) 22(2):6. 10.1007/s11926-020-0882-1 32020498

[11] Mammadov G Liu HH Chen WX Fan GZ Li RX Liu FF Hepatic dysfunction secondary to Kawasaki disease: characteristics, etiology and predictive role in coronary artery abnormalities. Clin Exp Med. (2020) 20(1):21–30. 10.1007/s10238-019-00596-1 31734766

[12] Liu X Wang L Shao S Zhang N Wu M Liu L Sterile pyuria in Kawasaki disease: a large prospective cohort study. Front Cardiovasc Med. (2022) 9:856144. 10.3389/fcvm.2022.856144 35647045 PMC9130598

[13] Choi JY Park SY Choi KH Park YH Lee YH. Clinical characteristics of kawasaki disease with sterile pyuria. Korean J Pediatr. (2013) 56(1):13–8. 10.3345/kjp.2013.56.1.13 23390440 PMC3564025

[14] Stehouwer CD Smulders YM. Microalbuminuria and risk for cardiovascular disease: analysis of potential mechanisms. J Am Soc Nephrol. (2006) 17(8):2106–11. 10.1681/ASN.2005121288 16825333

[15] Glassock RJ. Is the presence of microalbuminuria a relevant marker of kidney disease? Curr Hypertens Rep. (2010) 12(5):364–8. 10.1007/s11906-010-0133-3 20686930 PMC2941636

[16] Mahmoud HT Berton G Cordiano R Palmieri R Petucco S Bagato F. Microalbuminuria during acute coronary syndrome: association with 22-year mortality and causes of death. The ABC-8* study on heart disease. (*ABC is an acronym for Adria, Bassano, Conegliano, and Padova hospitals). Int J Cardiol. (2023) 374:100–7. 10.1016/j.ijcard.2022.12.025 36535560

[17] Yuan C Zhang Q. Risk factors for microalbuminuria in adult Tibetan patients with high-altitude pulmonary hypertension: a cross-sectional study. Cardiovasc Diagn Ther. (2023) 13(2):336–44. 10.21037/cdt-22-385 37583683 PMC10423733

[18] Ahmad T Ulhaq I Mawani M Islam N. Microalbuminuria in type-2 diabetes mellitus; the tip of iceberg of diabetic complications. Pak J Med Sci. (2017) 33(3):519–23. 10.12669/pjms.333.12537 28811763 PMC5510095

[19] Inoue K Streja E Tsujimoto T Kobayashi H. Urinary albumin-to-creatinine ratio within normal range and all-cause or cardiovascular mortality among U.S. Adults enrolled in the NHANES during 1999–2015. Ann Epidemiol. (2021) 55:15–23. 10.1016/j.annepidem.2020.12.004 33338645 PMC8202057

[20] Kovesdy CP Lott EH Lu JL Malakauskas SM Ma JZ Molnar MZ Outcomes associated with microalbuminuria: effect modification by chronic kidney disease. J Am Coll Cardiol. (2013) 61(15):1626–33. 10.1016/j.jacc.2012.11.071 23500283 PMC3625505

[21] Sung KC Ryu S Lee JY Lee SH Cheong E Hyun YY Urine albumin/creatinine ratio below 30 mg/g is a predictor of incident hypertension and cardiovascular mortality. J Am Heart Assoc. (2016) 5(9):e003245. 10.1161/JAHA.116.003245 27625343 PMC5079007

[22] Soeters PB Wolfe RR Shenkin A. Hypoalbuminemia: pathogenesis and clinical significance. JPEN J Parenter Enteral Nutr. (2019) 43(2):181–93. 10.1002/jpen.1451 30288759 PMC7379941

[23] Xia Y Qiu H Wen Z Shi H Yu H Li J Albumin level and progression of coronary artery lesions in Kawasaki disease: a retrospective cohort study. Front Pediatr. (2022) 10:947059. 10.3389/fped.2022.947059 36186633 PMC9516112

[24] McCrindle BW Rowley AH Newburger JW Burns JC Bolger AF Gewitz M Diagnosis, treatment, and long-term management of Kawasaki disease: a scientific statement for health professionals from the American Heart Association. Circulation. (2017) 135(17):e927–99. 10.1161/CIR.0000000000000484 28356445

[25] Rovin BH Adler SG Barratt J Bridoux F Burdge KA Chan TM Executive summary of the KDIGO 2021 guideline for the management of glomerular diseases. Kidney Int. (2021) 100(4):753–79. 10.1016/j.kint.2021.05.015 34556300

[26] Dallaire F Dahdah N. New equations and a critical appraisal of coronary artery Z scores in healthy children. J Am Soc Echocardiogr. (2011) 24(1):60–74. 10.1016/j.echo.2010.10.004 21074965

[27] Giannotti G Landmesser U. Endothelial dysfunction as an early sign of atherosclerosis. Herz. (2007) 32(7):568–72. 10.1007/s00059-007-3073-1 17972030

[28] Zhang D Liu L Huang X Tian J. Insights into coronary artery lesions in Kawasaki disease. Front Pediatr. (2020) 8:493. 10.3389/fped.2020.00493 32984207 PMC7477115

[29] Watanabe T Abe Y Sato S Uehara Y Ikeno K Abe T. Sterile pyuria in patients with Kawasaki disease originates from both the urethra and the kidney. Pediatr Nephrol. (2007) 22(7):987–91. 10.1007/s00467-007-0449-7 17323086

[30] Ohta K Seno A Shintani N Kato E Yachie A Seki H Increased levels of urinary interleukin-6 in Kawasaki disease. Eur J Pediatr. (1993) 152(8):647–9. 10.1007/BF01955240 8404968

[31] Azuma J Yamamoto T Nitta M Hasegawa Y Kijima E Shimotsuji T Structure equation model and neural network analyses to predict coronary artery lesions in Kawasaki disease: a single-centre retrospective study. Sci Rep. (2020) 10(1):11868. 10.1038/s41598-020-68657-0 32681105 PMC7368009

[32] Mahemuti N Zou J Liu C Xiao Z Liang F Yang X. Urinary albumin-to-creatinine ratio in normal range, cardiovascular health, and all-cause mortality. JAMA Netw Open. (2023) 6(12):e2348333. 10.1001/jamanetworkopen.2023.48333 38113044 PMC10731498

[33] Gerstein HC Mann JF Yi Q Zinman B Dinneen SF Hoogwerf B Albuminuria and risk of cardiovascular events, death, and heart failure in diabetic and nondiabetic individuals. JAMA. (2001) 286(4):421–6. 10.1001/jama.286.4.421 11466120

[34] Vart P Scheven L Lambers Heerspink HJ de Jong PE de Zeeuw D Gansevoort RT. Urine albumin-creatinine ratio versus albumin excretion for albuminuria staging: a prospective longitudinal cohort study. Am J Kidney Dis. (2016) 67(1):70–8. 10.1053/j.ajkd.2015.05.025 26188433

[35] de Boer RA Nayor M deFilippi CR Enserro D Bhambhani V Kizer JR Association of cardiovascular biomarkers with incident heart failure with preserved and reduced ejection fraction. JAMA Cardiol. (2018) 3(3):215–24. 10.1001/jamacardio.2017.4987 29322198 PMC5862778

[36] Kosmeri C Milionis H Vlahos AP Benekos T Bairaktari E Cholevas V The impact of dyslipidemia on early markers of endothelial and renal dysfunction in children. J Clin Lipidol. (2021) 15(2):292–300. 10.1016/j.jacl.2020.12.003 33478934

[37] Shah SJ Lam CSP Svedlund S Saraste A Hage C Tan RS Prevalence and correlates of coronary microvascular dysfunction in heart failure with preserved ejection fraction: pROMIS-HFpEF. Eur Heart J. (2018) 39(37):3439–50. 10.1093/eurheartj/ehy531 30165580 PMC6927847

[38] Pirro M Mannarino MR Francisci D Schiaroli E Bianconi V Bagaglia F Urinary albumin-to-creatinine ratio is associated with endothelial dysfunction in HIV-infected patients receiving antiretroviral therapy. Sci Rep. (2016) 6:28741. 10.1038/srep28741 27353425 PMC4926110

[39] Huang J Zhang S. Overexpressed neuropilin-1 in endothelial cells promotes endothelial permeability through interaction with ANGPTL4 and VEGF in kawasaki disease. Mediators Inflamm. (2021) 2021:9914071. 10.1155/2021/9914071 34434074 PMC8380503

[40] Lu Y Chen T Wen Y Si F Wu X Yang Y. Prediction of repeated intravenous immunoglobulin resistance in children with Kawasaki disease. BMC Pediatr. (2021) 21(1):406. 10.1186/s12887-021-02876-w 34530763 PMC8444587

[41] Li Y Zheng Q Zou L Wu J Guo L Teng L Kawasaki disease shock syndrome: clinical characteristics and possible use of IL-6, IL-10 and IFN-*γ* as biomarkers for early recognition. Pediatr Rheumatol Online J. (2019) 17(1):1. 10.1186/s12969-018-0303-4 30611297 PMC6321686

[42] Terai M Honda T Yasukawa K Higashi K Hamada H Kohno Y. Prognostic impact of vascular leakage in acute Kawasaki disease. Circulation. (2003) 108(3):325–30. 10.1161/01.CIR.0000079166.93475.5F 12835221

[43] Zheng X Li J Yue P Liu L Li J Zhou K Is there an association between intravenous immunoglobulin resistance and coronary artery lesion in Kawasaki disease?-current evidence based on a meta-analysis. PLoS One. (2021) 16(3):e0248812. 10.1371/journal.pone.0248812 33764989 PMC7993784

[44] Chbeir D Gaschignard J Bonnefoy R Beyler C Melki I Faye A Kawasaki disease: abnormal initial echocardiogram is associated with resistance to IV Ig and development of coronary artery lesions. Pediatr Rheumatol Online J. (2018) 16(1):48. 10.1186/s12969-018-0264-7 30021610 PMC6052519

[45] Hua W Ma F Wang Y Fu S Wang W Xie C A new scoring system to predict Kawasaki disease with coronary artery lesions. Clin Rheumatol. (2019) 38(4):1099–107. 10.1007/s10067-018-4393-7 30523553

[46] Tsai CM Yu HR Tang KS Huang YH Kuo HC. C-reactive protein to albumin ratio for predicting coronary artery lesions and intravenous immunoglobulin resistance in Kawasaki disease. Front Pediatr. (2020) 8:607631. 10.3389/fped.2020.607631 33324592 PMC7723900

[47] Muto T Masuda Y Nakamura N Numoto S Kodama S Miyamoto R Usefulness of brain natriuretic peptide to distinguish Kawasaki disease from cervical lymphadenitis. Pediatr Int. (2022) 64(1):e15050. 10.1111/ped.15050 34739174

[48] Kobayashi T Ayusawa M Suzuki H Abe J Ito S Kato T Revision of diagnostic guidelines for Kawasaki disease (6th revised edition). Pediatr Int. (2020) 62(10):1135–8. 10.1111/ped.14326 33001522

